# The value of local consolidative therapy in Osimertinib-treated non-small cell lung cancer with oligo-residual disease

**DOI:** 10.1186/s13014-020-01651-y

**Published:** 2020-08-27

**Authors:** Ya Zeng, Jianjiao Ni, Fan Yu, Yue Zhou, Yang Zhao, Shuyan Li, Tiantian Guo, Li Chu, Xi Yang, Xiao Chu, Xuwei Cai, Zhengfei Zhu

**Affiliations:** 1 Department of Radiation Oncology, Fudan University Shanghai Cancer Center, Shanghai, 200032 China; 2 Department of Radiation Oncology, Shanghai Chest Hospital, Shanghai Jiao Tong University, Shanghai, 200030 China; 3 Department of Oncology, Shanghai Medical College, Fudan University, Shanghai, 200032 China

**Keywords:** Non-small cell lung cancer, Osimertinib, Oligo-residual disease, Local consolidative therapy

## Abstract

**Background:**

There was no study investigating real-world utilization and outcome of LCT in Osimertinib-treated NSCLC with oligo-residual disease. This study was to analyze the clinical value of local consolidative therapy (LCT) in Osimertinib-treated non-small cell lung cancer (NSCLC) patients with oligo-residual disease.

**Methods:**

Patients receiving standard Osimertinib treatment and developing oligo-residual disease (five or fewer residual metastatic lesions) were retrospectively reviewed. Local therapies performed to the oligo-residual tumor lesions or primary lung site before Osimertinib treatment failure were considered as LCT.

**Results:**

Of 108 patients recruited, first-line and second-line Osimertinib were administered in 25 and 83 patients, respectively, while LCT was performed in 14 patients. With a median follow-up of 43.6 months, 69 patients developed progressive disease. LCT significantly improved progression-free survival (PFS) (NR vs 12.8 months, *p* = 0.01) and was independently associated with prolonged PFS (HR = 0.29, 95%CI 0.12 to 0.68, *p* = 0.004). Patients receiving LCT had a numerically longer overall survival (OS) (85.8 vs 77.1 months, *p* = 0.58) and after adjusting for potentially confounding factors, LCT was associated with a non-significantly prolonged OS (HR = 0.37, 95%CI 0.12–1.16, *p* = 0.089). Pattern of failure analyses indicated that progressive disease developed at the originally existed oligo-residual lesions in 76.2% of the 63 patients who didn’t receive LCT and had Osimertinib treatment failure. Of note, 7 (70%) of the 10 patients who had oligo-residual cranial disease but didn’t receive LCT, developed more than five progressive lesions in the brain, which were no longer suitable for stereotactic radiosurgery.

**Conclusion:**

Among Osimertinib-treated NSCLC patients having oligo-residual lesions, LCT could improve local control and significantly increase PFS, which need to be verified by further investigations.

## Background

Epidermal growth factor receptor (EGFR) tyrosine kinase inhibitors (TKIs) are the standard first-line therapy for advanced non-small cell lung cancer (NSCLC) patients harboring EGFR-sensitizing mutations [1]. There is an inevitable fact that, however, most patients would ultimately suffer disease progression [2–4]. Acquired EGFR ThrT790Met resistance mutation (T790M) appeared frequently in over half of patients who received first- or second-generation EGFR-TKIs [5–7].

Osimertinib is an oral, third-generation, irreversible EGFR-TKI that was proved to selectively inhibit both EGFR-TKI-sensitizing and EGFR T790M resistance mutations [8, 9]. Osimertinib has been the standard treatment for patients with metastatic T790M-positive NSCLC that progressed from EGFR-TKI treatment based on the AURA3 clinical trial with an impressive PFS extension [10–12]. It was also approved to be one of the first-line treatment options for EGFR-mutant NSCLC patients owing to the positive results from the FLAURA study, which demonstrated significant survival benefits in both PFS and OS [13, 14].

Accumulating evidence suggests that local consolidative therapy (LCT), such as surgery, radiotherapy and radiosurgery, could improve survival in highly selected patients with advanced NSCLC who have disease control after initially systemic therapy [15–19]. The landmark multicenter phase II study showed that LCT after effective systemic therapy significantly improved patients’ PFS and OS in oligometastatic NSCLC, when compared with conventional maintenance therapy [18, 19]. A retrospective study conducted by Xu et al., including synchronous oligometastatic EGFR-mutant NSCLC treated with first-generation EGFR TKIs, revealed that LCT administered to extracranial lesions and/or cranial lesions improved both PFS and OS. Meanwhile, in the prospective ATOM study, pre-emptive local therapy performed by stereotactic ablative radiotherapy, was feasible and prolonged PFS in first- or second-generation EGFR TKI treated NSCLC with oligo-residual disease. However, patients recruited in the studies mentioned above all received LCT when patients were treated with first- or second-generation EGFR-TKIs [15, 16]. It is well known that Osimertinib has higher potency against both cranial and extracranial tumor lesions, when compared with first- or second-generation EGFR TKIs. However, acquired resistance to Osimertinib was also inevitable [20, 21], and the clinical values of LCT in the era of Osimertinib for EGFR-mutant NSCLC remained unknown.

Our previous study found that 26.8% of EGFR-mutant NSCLC patients treated with Osimertinib were suitable for consolidative stereotactic body radiotherapy (SBRT) at the time of maximal response to Osimertinib [22]. However, there was no study investigating real-world utilization and outcome of LCT in Osimertinib-treated NSCLC with oligo-residual disease. Herein, we retrospectively examined the survival outcomes and patterns of treatment failure in Osimertinib-treated NSCLC patients with oligo-residual disease, receiving LCT or not in two academic centers, in order to determine the clinical values of LCT in such patients.

## Methods

### Patients

**Fig. 1:**
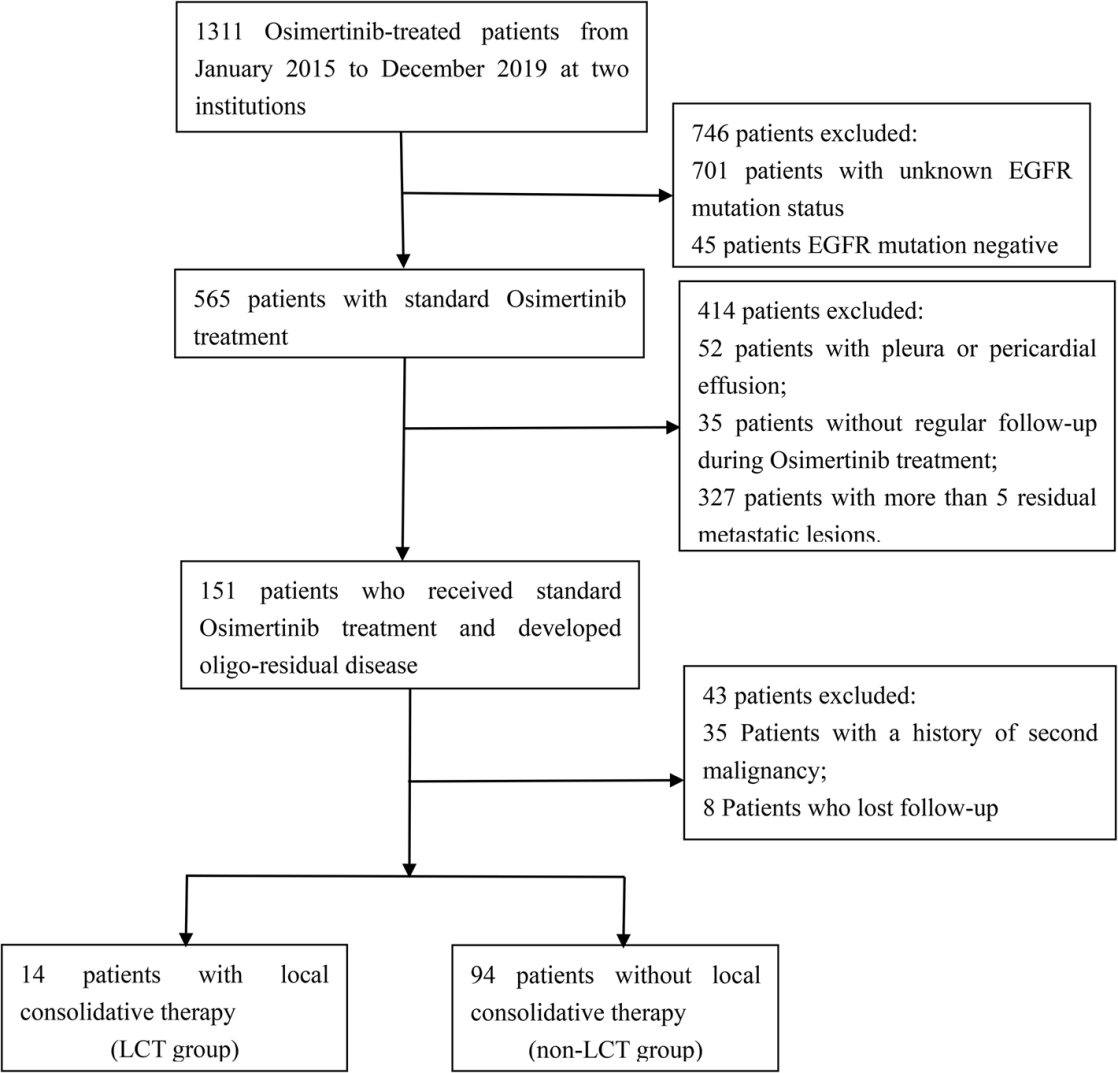
Patients enrollment flowchart

Patients with EGFR-mutant advanced NSCLC who received standard Osimertinib treatment in clinical trials or routine practice from January 2015 to December 2019 at Fudan University Shanghai Cancer Center and Shanghai Chest Hospital, were retrospectively reviewed. The inclusion criteria were as follows: 1) patients with pathologically diagnosed EGFR-mutant advanced NSCLC; 2) receiving standard Osimertinib treatment (first-line Osimertinib in untreated patients or second-line Osimertinib in pretreated T790M-positive patients); 3) having oligo-residual disease, which was defined according to the consensus of oligometastatic disease [23], during Osimertinib treatment with five or fewer residual metastatic lesions, excluding primary lung tumor (cranial and lymph node metastasis were allowed [16], and counted per lesion). The exclusion criteria included: 1) patients with a history of second malignancy; 2) patients with pleural or pericardial effusion; 3) patients without adequate follow-up information to determine the status of residual disease. The patients’ selection flowchart was shown in Fig. 1. Both Fudan University Shanghai Cancer Center and Shanghai Chest Hospital Institution Review Board approved this study. Informed consent was waived by the institutional review boards because this was a retrospective study.

### Treatment and follow-up

Patients received Osimertinib with a standard dosage of 80 mg/day as a sole systemic therapy were included in this study. LCT, including surgery, radiotherapy, and radiosurgery, were performed during Osimertinib treatment and to the primary lung tumors or the metastatic lesions, to the extracranial lesions or the intracranial metastases, in some of the patients. Generally, there would be a multidisciplinary team to decide whether the patient with oligo-residual disease needed to receive LCT. The final decision was at the discretion of the treating physician, as well as patient’s preference.

Patients were generally followed up every 2 months. Chest computed tomography (CT) scans, CT scan or ultrasonography of abdominal and cervical regions, were routinely performed. Brain magnetic resonance imaging, positron emission tomography and bone scanning were not mandatory, and were performed at the discretion of the treatment physicians. Serial imaging of each patient was reviewed by a senior radiologist. Telephone calls were also implemented when necessary.

### Statistical methods

The overall survival (OS) was defined as the time from the date of diagnosis to death of any causes. Progression-free survival (PFS) was defined as the time from the date of the initiation of Osimertinib to the date of disease progression (by Response Evaluation Criteria in Solid Tumors [RECIST], version 1.1) or death of any causes. Patients who lived without disease progression were recorded as censored. OS and PFS were estimated by Kaplan-Meier method. Log-rank tests was used to compare the survival curves. Cox proportional hazards regressions were used to evaluate prognostic factors and calculate hazard ratios (HRs) for OS and PFS. *P* value less than 0.10 of clinical characteristics in univariate analyses and LCT were forcedly included in the multivariate analyses for OS owing to the small sample size. *P* value less than 0.05 (two sided) was considered statistically significant in this study.

## Results

### Patients characteristics

There were 108 Osimertinib-treated advanced NSCLC patients recruited in this study, 14 of whom underwent LCT and the other 94 did not. There were 18.1% (17/94) of patients in non-LCT group and 8 patients in LCT group received Osimertinib as first-line treatment. Local consolidative therapy was performed at a median time of 2.2 months (range, 1.5–10.0 months) after Osimertinib initiation. Three patients received surgical resection (brain = 1, lung = 2), 3 received cranial radiotherapy (stereotactic radiosurgery = 1, whole brain radiotherapy = 2) and the other 8 received ablative extracranial radiotherapy (lung = 4, lymph node = 3, bone = 1). Among the 4 patients receiving irradiation to the lung, stereotactic body radiotherapy was performed in 3 patients. The clinical characteristics of the enrolled patients at the time of developing oligo-residual disease were presented in Table 1. Patients with less metastatic lesions (*p* = 0.018), receiving first-line Osimertinib treatment (*p* = 0.003) and without lung metastasis (*p* = 0.006), were more likely to receive LCT.

**Table 1:** Disease characteristics of patients in non-LCT group and LCT group

	no-LCT (94)	LCT (14)	*p* value
Sex			0.63
female	54(57.4%)	9(64.3%)	
male	40(42.6%)	5(35.7%)	
Age (years)			0.36
Median (range)	62(38–83)	61(35–70)	
Smoking			0.29
no	53(56.4%)	10(71.4%)	
yes	41(43.6%)	4(28.6%)	
T stage			0.30
T0–2	78(83.0%)	13(92.9%)	
T3–4	16(17.0%)	2(7.1%)	
N stage			0.36
N0	48(51.1%)	9(64.3%)	
N1–3	46(48.9%)	5(35.7%)	
EGFR mutation			0.49
19del	45(47.9%)	7(50.0%)	
L858R	44(46.8%)	7(50.0%)	
others	5(5.3%)	0(0.0%)	
No. mets			0.018
≤ 2	42(44.7%)	11(78.6%)	
> 2	52(55.3%)	3(21.4%)	
No. mets. Organs			0.13
≤ 2	86(91.5%)	14(100%)	
> 2	8(8.5%)	0(0.0%)	
Lung mets			0.006
no	31(33.0%)	10(38.0%)	
yes	63(67.0%)	4(28.6%)	
Bone mets			0.51
no	66(70.2%)	11(78.6%)	
yes	28(29.8%)	3(21.4%)	
Adrenal gland mets			1.00
no	89(94.7%)	14(100%)	
yes	5(5.3%)	0(0.0%)	
Brain mets			0.07
no	64(68.1%)	6(42.9%)	
yes	30(31.9%)	8(57.1%)	
LN mets			0.29
no	53(56.4%)	10(71.4%)	
yes	41(43.6%)	4(28.6%)	
Osimertinib			0.003
First-line	17(18.1%)	8(57.1%)	
Second-line	77(81.9%)	6(42.9%)	

Abbreviations: *LCT* local consolidative therapy, *No* number, *mets* metastasis, *LN* lymph node

### Survival outcomes

**Fig. 2:**
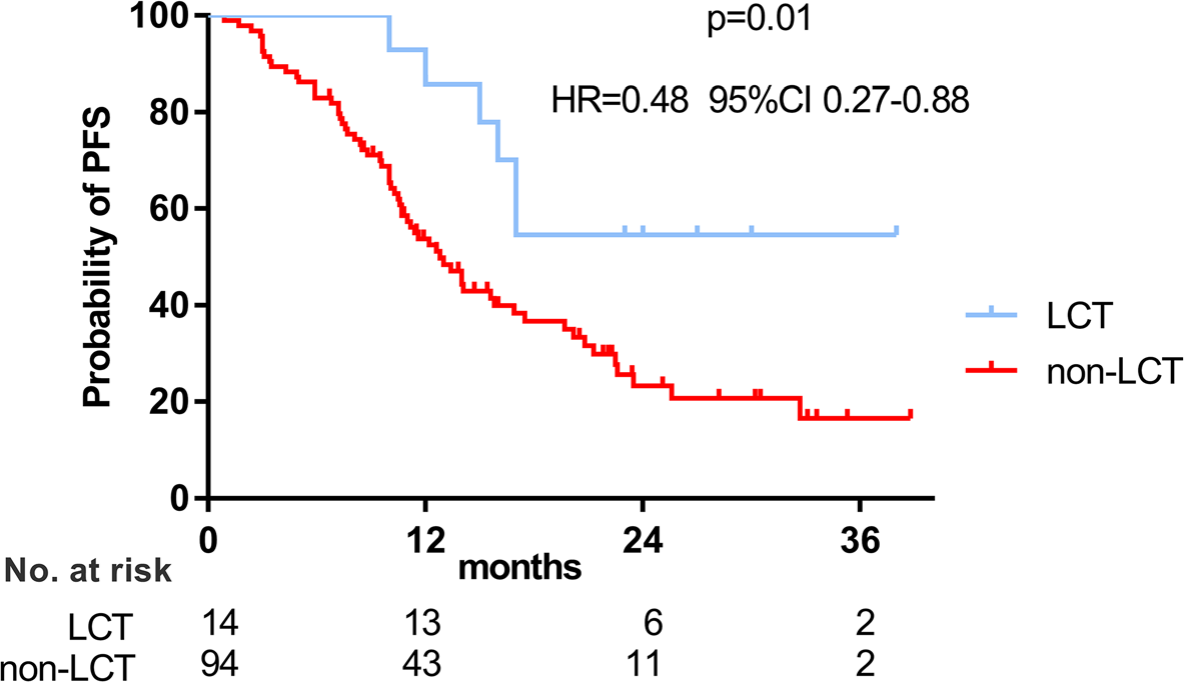
Progression-free survival (PFS) curves of LCT group and non-LCT group. The median PFS of LCT group was not reached and 12.8 months for patients in non-LCT group. LCT: local consolidative therapy

With a median follow-up of 43.6 months (range, 9.3–114.1 months), 69 patients developed progressive disease. The median progression-free survival (PFS) of the whole cohort was 14.0 months. The median PFS of patients in the non-LCT group was 12.8 months and the median PFS of patients in the LCT group was not yet reached. 1-year and 3-year PFS rate were 85.7, 54.5% for patients in the LCT group and 53.7, 16.6% for patients in the non-LCT group, respectively. The difference of PFS between two groups was statistically significant (*p* = 0.01, HR = 0.48, 95%CI 0.27 to 0.88, Fig. 2). Sex (*p* = 0.02, HR = 2.71, 95%CI 1.14–6.42), T stage (*p* = 0.01, HR = 1.30, 95%CI 1.06–1.59) and LCT (*p* = 0.004, HR = 0.29, 95%CI 0.12–0.68) were found to be independent predictors of PFS (shown in Table 2).

**Table 2:** Univariate and multivariate analysis of progression-free survival

	Univariate analysis		Multivariate analysis	
	HR (95%CI)	*p* value	HR (95%CI)	*p* value
sex	1.79(1.12–2.89)	0.02	2.71(1.14–6.42)	0.02
Age (years)	0.84(0.52–1.35)	0.47		
smoking	1.61(0.99–2.59)	0.05	0.59(0.24–1.44)	0.25
T stage	0.35(0.20–0.63)	< 0.001	1.30(1.06–1.59)	0.01
N stage	0.95(0.59–1.52)	0.82		
EGFR mutation	0.94(0.62–1.44)	0.78		
No. mets	0.92(0.57–1.47)	0.72		
No. mets. Organs	0.51(0.22–1.18)	0.12		
lung mets	1.26(0.77–2.06)	0.37		
bone mets	1.55(0.94–2.55)	0.08		
adrenal gland mets	2.49(0.90–6.91)	0.08		
brain mets	1.38(0.85–2.27)	0.19		
LN mets	1.14(0.70–1.84)	0.60		
Osimertinib	0.73(0.42–1.27)	0.27		
LCT	0.48(0.27–0.88)	0.02	0.29(0.12–0.68)	0.004

Abbreviations: *LCT* local consolidative therapy, *No* number, *mets* metastasis, *LN* lymph node

**Fig. 3:**
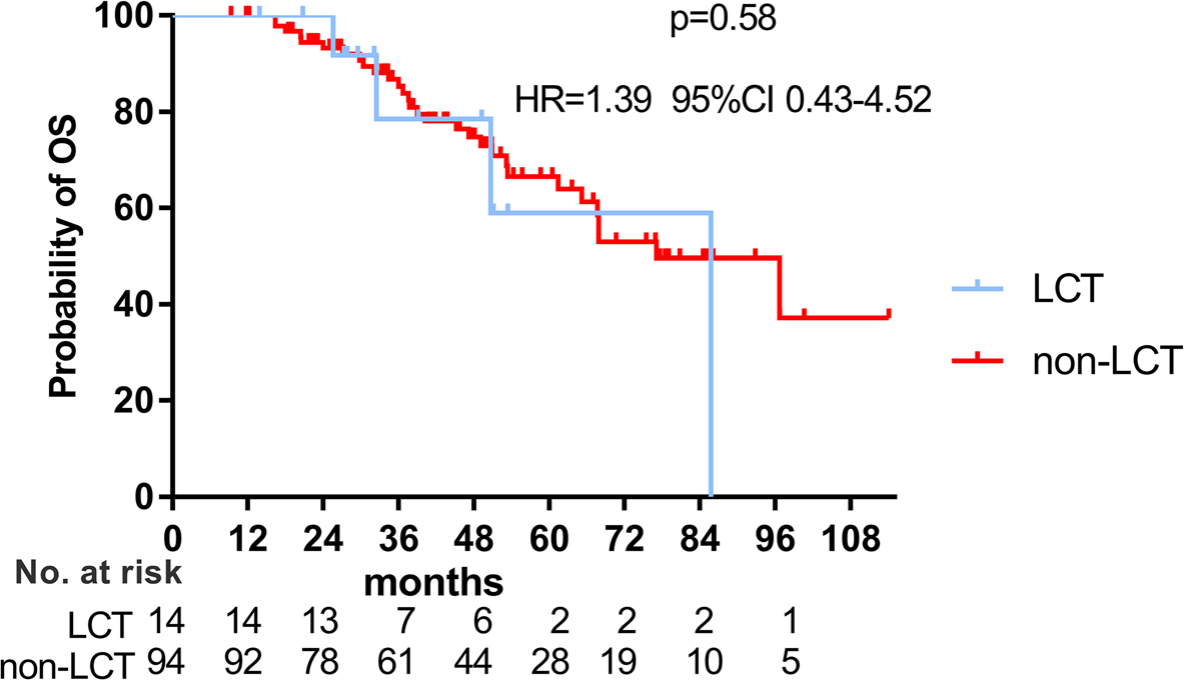
Overall survival (OS) curves of LCT group and non-LCT group. The median OS were respectively 85.8 months and 77.1 months for patients in LCT group and non-LCT group. LCT: local consolidative therapy

By the time of data-cut off, 34 patients had died and the median OS was 77.1 months. The 1-, 3-, 5-year OS rate were 96.1, 84.7 and 65.8%, in the entire cohort, retrospectively. Patients receiving LCT had a numerically longer overall survival (OS) (85.8 vs 77.1 months, *p* = 0.58**,** Fig. 3) and after adjusting potential confounding factors using Cox analyses, LCT was associated with a non-significantly prolonged OS (HR = 0.37, 95%CI 0.12–1.16, *p* = 0.089) (shown in Table 3).

**Table 3:** Univariate and multivariate analysis of overall survival

	Univariate analysis		Multivariate analysis	
	HR (95%CI)	*p* value	HR (95%CI)	*p* value
sex	1.78(0.90–3.52)	0.09	1.30(0.62–2.73)	0.49
age (years)	0.66(0.33–1.32)	0.24		
smoking	1.45(0.71–2.89)	0.29		
T stage	0.27(0.12–0.57)	0.001	0.29(0.12–0.70)	0.006
N stage	0.53(0.26–1.06)	0.07	0.55(0.26–1.16)	0.12
EGFR mutation	1.25(0.67–2.34)	0.48		
No. mets	0.99(0.51–1.99)	0.99		
No. mets. Organs	0.27(0.09–0.77)	0.02	1.01(0.27–4.30)	0.92
lung mets	1.91(0.89–4.10)	0.09	2.0(0.72–6.99)	0.11
bone mets	0.95(0.46–1.95)	0.89		
adrenal gland mets	1.94(0.46–8.17)	0.35		
brain mets	1.68(0.84–3.36)	0.14		
LN mets	2.05(1.02–4.10)	0.04	1.80(0.82–3.96)	0.15
Osimertinib	1.07(0.31–3.71)	0.91		
LCT	1.39(0.43–4.52)	0.58	0.37(0.12–1.16)	0.09

Abbreviation: *LCT* local consolidative therapy, *No* number, *mets* metastasis, *LN* lymph node

### Patterns of treatment failure

There were 67% (63/94) of patients in non-LCT group suffered Osimertinib treatment failure. Among them, 55.6% (35/63) of patients developed progressive disease only at the originally existed residual lesions (termed as original failure), 23.8% (15/63) of patients developed progressive disease only at distant new sites (termed as distant failure) and the rest (20.6%) patients developed progressive disease at both sites (termed as mixed failure). The most common sites of progressive disease were brain (21.6%), lung (16.2%), bone (16.2%) and lymph node (14.9%). Additionally, 27.0% (17/63) of patients with progressive disease received certain kind of salvage local therapy. Of note, 21 of the 30 patients with oligo-residual cranial lesions who didn’t receive LCT had progressive disease, 10 of whom developed progressive disease in the brain. Moreover, 7 of the 10 patients developed more than 5 progressive cranial lesions after Osimertinib treatment failure. Salvage brain radiotherapy were performed in 4 of the 7 patients, all of which were whole brain radiotherapy (WBRT).

**Fig. 4:**
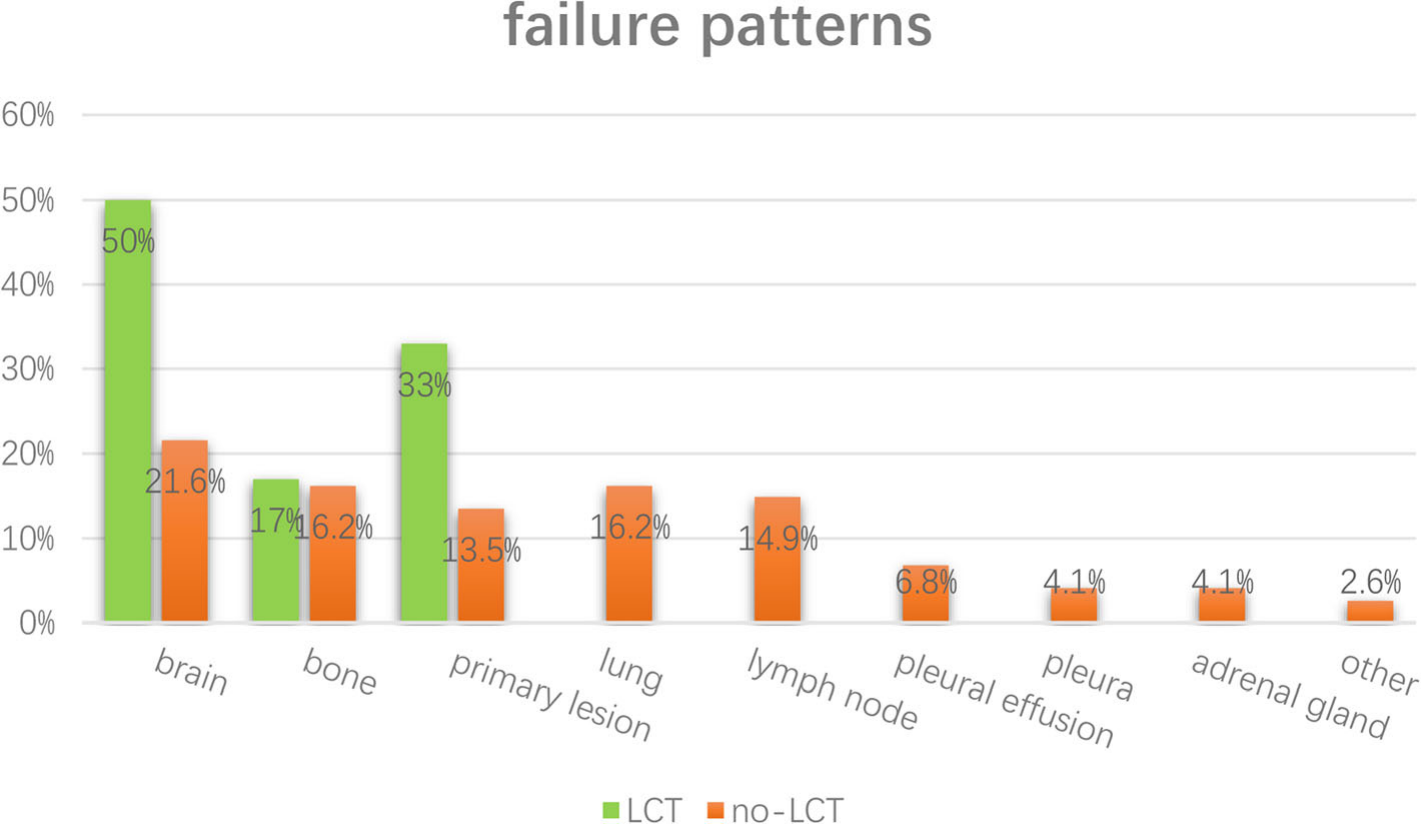
The details of failure patterns of two groups. LCT: local consolidative therapy

There were totally 6 patients in the LCT group suffered failure. The failure patterns included brain (3/6 patients), bone (17%) and primary lesion (33%). 4 of 6 patients were original failure and the others were mixed failure. The details of the patterns of treatment failure for two groups were shown in Fig. 4.

## Discussion

As far as we know, this was the first study that explored the real-world utilization and outcome of LCT in Osimertinib-treated NSCLC patients with oligo-residual disease. We found that LCT could significantly improve PFS in such patients. The pattern of failure analyses also favored administration of LCT instead of salvage local therapy, especially for those with oligo-residual cranial lesions, since deferring brain radiation may make these patients losing the opportunity of stereotactic radiosurgery (SRS), which has been demonstrated to have less neuro-toxicities compared with WBRT.

The rationale and feasibility of LCT in Osimertinib-treated NSCLC are validated in the current study. First, 55.6% (35/63) of patients in the non-LCT group developed progressive disease in the originally existed oligo-residual tumor sites, which was consistent with previous studies [24–26] and strongly indicated a potentially beneficial role of LCT. Second, 151 out of the 565 (26.7%) Osimertinib-treated NSCLC patients were identified to harboring oligo-residual disease in our study and it validated our previous result generated from Osimertinib-treated patients cohort with a smaller sample size, among which 26.8% of patients were found to be suitable for consolidative stereotactic body radiotherapy (SBRT) at the time of maximal response to Osimertinib [22]. Taken together, these data suggested that LCT, to part or all of the residual sites, could be performed in a considerable percentage of Osimertinib-treated NSCLC patients. In the current study, about 10% of the potential candidates received certain kind of LCT uneventfully, preliminarily demonstrated the feasibility and safety of LCT in such patients.

LCT to oligo-residual sites could significantly improve the PFS for EGFR-mutant NSCLC treated with EGFR-TKIs. Previous studies have found that LCT may prolong PFS in first- or second-generation EGFR-TKI treated NSCLC with oligometastatic disease by 4–6 months [15, 16, 27]. LCT, for the first-time, was shown to significantly prolong PFS in Osimertinib-treated NSCLC in our study. The median PFS for patients treated with Osimertinib alone was 12.8 months in our study, which was within the reasonable range since patients receiving first-line or second-line Osimertinib were both included [11, 16]. Meanwhile, in the retrospective study which included patients with stage IV EGFR-mutant NSCLC who had oligometastatic disease during first-line EGFR-TKI therapy, Xu et al. implied that only LCT to all sites can prolong PFS [16]. The interim results of the randomized phase III, open-label SINDAS trial showed that upfront stereotactic radiotherapy (SBRT) delivered to all of the oligo-metastatic sites in combination with first-line EGFR-TKI significantly improved both PFS and OS compared with EGFR-TKI alone [28]. In our study, LCT performed either to part of the residual sites or to all of the residual sites, analyzed together, were found to significantly decrease the risk of disease progression, when compared with those received no LCT (HR = 0.48, 95%CI, 0.27 to 0.88). Due to the limited sample size, we could not further examine the separate role of LCT performed to part of the residual sites and those performed to all of the residual sites. Whether LCT to all oligometastatic sites could bring further survival benefit to patients with Osimertinib needs to be further investigated.

Patients in LCT group had a numerically longer survival than that of non-LCT group, but the improvement did not reach statistical significance in this study. There were a few studies implied that LCT could improve OS of patients treated with first generation EGFR-TKIs. Hu et al. performed a study retrospectively recruiting 231 patients and found that LCT plus EGFR-TKI for patients with oligometastatic disease could significant improve OS compared with EGFR-TKI monotherapy alone (34 months vs 21 months) [15]. Another retrospective study conducted by Xu et al. also revealed a statistical improvement of median OS by 10.1 months [16]. In the SINDAS trial, upfront SBRT combined with first-line EGFR-TKI significantly improved OS by 8.1 months compared with EGFR-TKI alone [28]. In the current study, however, the numerical improvement of OS for patients in LCT group did not reach statistical significance. The limited sample size of the study could be the main reason. Meanwhile, 27% patients in the non-LCT group received salvage local treatment when disease progressed after Osimertinib, which may be another confounding factor that may influence the OS result. As the Swiss cohort study discovered that salvage local therapy improved OS in Osimertinib-treated NSCLC with oligo-progressive disease [25]. Moreover, some of the patients in our study received LCT performed to part but not all of the residual sites and this may weaken the magnitude of clinical benefit of LCT in such patients, which had been demonstrated by the study conducted by Xu et al. [16]. What we need to state was that the median OS was markedly longer in our study (LCT group vs. non-LCT group: 85.8 months vs. 77.1 months) than previous studies [16, 27]. For example, in the double-blind, randomized phase 3 FLAURA trail, a mean OS of 38.6 months were reported among patients receiving first-line Osimertinib [14]. One of the main reasons to explain the extraordinary long OS in our study was that most of the patients received second-line Osimertinib and OS was calculated from the diagnosis of advanced NSCLC. One previous study found that among patients who failed former first- or second-generation EGFR TKIs and acquired EGFR T790M mutation, Osimertinib treatment could induce a median OS of 50.4 months [29]. In addition, patients in this study were all harboring oligo-residual disease, whom are generally having more indolent disease and could have a relative longer overall survival [30]. Advanced NSCLC patients receiving curative treatment approaches for metastatic sites [31] had an obviously longer 5-year OS rate than those treated with palliative intent [32].

The optimal timing of local therapies for patients with EGFR-TKI treated NSCLC remains controversial [17, 18, 33]. We support the utilization of LCT to the oligo-residual disease rather than salvage local therapy to the oligo-progressive disease. Patients who received LCT to oligo-residual sites had less and smaller lesions [15, 16, 34] than patients who received salvage local therapy with oligo-progression disease. And the corresponding toxicities might be lower, which was partially supported by the phase II study exploring the efficacy of LCT to oligo-residual lesions after TKIs treatment [27]. Furthermore, due to the potent efficacy of Osimertinib in patients with brain metastasis [9], oligo-residual cranial disease at the maximal response of Osimertinib was not uncommon, which might be suitable for SRS. Whereas multiple progressive disease may develop in the central nervous system after Osimertinib treatment failure, where salvage WBRT is needed [33, 35]. And thus, deferring local cranial local therapy until Osimertinib treatment may make some patients lose the valuable opportunity of the less-toxic SRS. In a word, LCT may bring certain benefit for oligo-residual NSCLC patients treated with Osimertinib. While, there was an urgent need to recruit more patients to analyze whether patients with Osimertinib therapy could gain a statistical OS improvement from LCT in the future.

Given the small number of patients and the retrospective nature of the current study, there are some limitations. First, selection bias apparently existed which led to the imbalance of disease characteristics between the two groups, although Cox proportional hazards regressions were employed in order to reduce the possible bias. The results needed to be interpreted with caution. Second, LCT was performed to part of the oligo-residual disease, but not all of the oligo-residual disease, in most of the patients in the present study. This may also lead to the result that LCT failed to significantly improve OS. Lastly, as a retrospective study, we failed to obtain adequate data to analyze the toxicities of LCT.

## Conclusions

LCT could significantly improve local control and PFS in Osimertinib-treated NSCLC patients with oligo-residual disease, which need to be verified by prospective study with a larger sample size.

## Data Availability

All data generated or analyzed during this study are included in this published article.

## Abbreviations

NSCLC: Non-small cell lung cancer
LCT: Local consolidative therapy
PFS: Progression-free survival
OS: Overall survival
EGFR-TKI: Epidermal growth factor receptor tyrosine kinase inhibitors
CT: Chest computed tomography
HRs: Hazard ratios
SRS: Stereotactic radiosurgery
SBRT: Stereotactic body radiotherapy
WBRT: Whole brain radiotherapy
Mets: Metastasis
No: Number
LN: Lymph node
